# Editorial: Understanding Young Individuals' Autonomy and Psychological Well-Being

**DOI:** 10.3389/fpsyg.2021.750115

**Published:** 2021-09-23

**Authors:** Teresita Bernal-Romero, Miguel Melendro, Ángel De-Juanas, Martin Goyette

**Affiliations:** ^1^Faculty of Psychology, Santo Tomás University, Bogotá, Colombia; ^2^Faculty of Education, National University of Distance Education (UNED), Madrid, Spain; ^3^École Nationale d'administration Publique, Quebec City, QC, Canada

**Keywords:** adolescents, psychological well-being, autonomy, youth—young adults, education

During the transitional stage between adolescence and adulthood, young people navigate complex and often difficult situations as they attain full maturity and autonomy. They strive to create their identities, defend their ideals, make decisions, and establish a sense of independence that will be crucial to their confidence about providing for themselves, taking responsibility for their actions, and handling the problems that will arise. Although young people feel a pressing need for self-fulfillment and independent living away from their parents, and for forging bonds with their peers, multiple studies have shown that autonomy develops gradually over time. Parents, educators, psychologists, and social workers are consequently very interested in exploring the factors affecting autonomy in young people, for purposes of developing counseling, orientation, and intervention strategies.

The autonomy and well-being of young people transitioning to adulthood is in fact very important in psychological, educational, and social research. Large quantities of empirical data are to be found within the framework of positive psychology; these data examine perceived psychological well-being compared to academic performance, personality, intelligence, etc.

Several interesting studies from psychological, educational, and social perspectives have examined autonomy processes in young people (here, ‘autonomy’ refers to the capacities for self-organization, context analysis, critical thinking, forming relationships with others, and socio-political engagement).

However, very few studies have linked psychological well-being to autonomy. The articles in this collection, addressing autonomy and psychological well-being in young people, all examine the link between these two variables and their relationships with other variables (peer groups, gender, physical activity, hobbies, geographic setting, etc.) that affect the lives of young people in our modern societies.

This topic is presented with three aims: (1) to discover new relationships, which may help explain a range of realities in different cultural and socio-educational contexts; (2) to deepen our understanding of the methodology used to examine and analyze these variables; and (3) to develop tested and effective plans of action, whether psychological, educational, or social, for the youth population.

The articles in this collection, which derive from different approaches and fields, therefore contribute to our understanding of autonomy and well-being in young people transitioning to adult life. This Research Topic is presented in order to promote new findings from original studies and reviews that provide a better overview of this reality and how it is affected by psychological, educational, and social interventions.

We have identified three lines of research among the articles contributing to this Research Topic: (1) a line examining the different *contextual approaches* to development, according to the most general understanding of the link between autonomy and psychological well-being; (2) a line addressing different *specific factors* and their impact on this area of knowledge examples include ICTs, physical activity/sports, and factors linked to gender and leisure/free time; and (3) a final line of research studying autonomy and well-being in adolescents and young adults within *educational systems*, including universities.

Beginning with the line of research exploring the different *contextual approaches* that are applied according to the most general understanding of the relationship between autonomy and psychological well-being in youth, we first highlight the creation and validation of an Autonomy Scale for young people transitioning to adulthood. This scale, devised by Bernal-Romero et al. employs a differential approach to measuring autonomy in young people. While most existing scales are based on a self-focused notion of autonomy, the scale presented in the above article contemplates the idea of the individual as mediated by others, and by society, along four dimensions: self-organization, context analysis, critical thinking, and socio-political engagement.

Another interesting contribution to this line of research, by Gabriel et al. examines the impact of experiences in a residential facility among young people leaving care. The authors gauge this impact in three analytic areas: social networks, parenthood, and state interventions. In a third article, Melendro et al. compare autonomy and social well-being in young people transitioning to adulthood by means of three different pathways, characterized by education, by employment and job training, or by extreme social disadvantage, with this last pathway displaying more complex situations. The study on group characteristics among adolescents and young adults facing social disadvantages or displaying maladjusted behavior, in the words of Bojanowska and Piotrowski, plus an interesting article on the effect of age on the development of autonomy and social well-being among young people (García-Castilla et al.), also provide helpful information for understanding these realities and designing evidence-based interventions for young people transitioning to adult life.

The line of research exploring contextual approaches also includes three studies that examine relevant problems in several other contexts informing how we understand and interact with young people in social difficulty: the influence of drugs (Santibáñez et al.), the effect of socioeconomic inequality on mental health (Myhr et al.), and how incarceration affects young people's development. In this third study, Añaños et al. found that assuming responsibilities, engaging in teamwork, feeling prepared for employment, and having an optimistic view of the future were positive factors contributing to readiness for temporary release. An additional study by Charry et al. compares Spanish and Colombian youth and examines the importance of contextual factors, finding significant differences in results for some dimensions of autonomy (self-organization and critical thinking) and of psychological well-being (the “positive relationships” and “purpose in life” subscales).

The second line of research examines the relationship between young people's autonomy and psychological well-being and different study variables having to do with leisure activity; this collection includes articles examining use of the Internet and social networks, physical activity and sports, and relationship status. These studies clearly show that leisure activities and hobbies remain one of the largest socio-educational and psychological concerns of our time. Increased free time with an expanded list of possibilities for leisure activities is not always conducive to best practices in our society (Audrin and Blaya). Social networks play a key role in these activities, as stated by Castillo de Mesa et al. Nonetheless, we must stress the social, educational, and psychological value of young people's leisure activities for their development and well-being. Findings from the articles by Rodríguez-Bravo et al.; Fraguela-Vale et al. support this position.

One must also consider the importance of leisure activities for optimal human development. They hold undeniable social, educational, and psychological value by forging intergenerational family relationships, as concluded by Alonso Ruiz et al. The articles by García-Castilla et al. and by Mari-Ytarte et al. both focus on peer relationships in young people and conclude that relationship status and sex education affect youth autonomy and psychological well-being. In any case, skillful time management is key to achieving a more satisfactory experience with either relationships or activities, according to findings by both Doistua et al.; Pestana et al.

A third and final line of research addresses the autonomy and well-being of adolescents and young people in educational systems, including universities. The first cluster of articles examines the importance of psychological well-being in the education of adolescents. In one study, framed from the parallel mediating model, Guo et al. show how teacher support may promote mental well-being in adolescents by increasing goal planning, affect control, and help-seeking behavior, while decreasing depression. Another study by Páez-Gallego et al., examining decision-making processes in adolescent students, found that increased use of adaptive decision-making strategies was correlated with higher levels of psychological well-being. Also within this cluster of articles, the study by Lan and Zhang on emotional well-being among Chinese adolescents who change schools shows how such disruptions can be detrimental to students' emotional state. It also addresses the role of professors in supporting student autonomy.

Last of all, this collection presents four articles focusing on university students. The first, by González-Olivares et al., explores the interaction between psychological well-being and motivation. It shows how the vocational approach in university teacher training programs provides new students with the motivation to achieve a professional future. Another two articles examine autonomy processes. Valenzuela et al., have linked procrastination with self-regulation profiles in their study of autonomous functioning in students. In the other, Borjas et al., examined financial independence and its effect on academic performance, finding lower scores among independent students who self-financed their studies or worked during the week than among students receiving loans or grants.

The final article, which actually coincided with the pandemic that provided the setting for the development of this Research Topic, was published by Fernández-Cruz et al. and focuses on evaluating emotional and cognitive regulation among young people in lockdown due to Covid-19. One of its more interesting findings is that the students' digital competence and ability to engage in online interactions were crucial for overcoming feelings of loneliness and social isolation, even when students were physically separated from their friends and classmates for a time.

The full set of studies listed in this collection clearly provides a very complete and diverse view of how we understand young people's autonomy and psychological well-being, while specifically focusing on those people facing greater social difficulties. This Research Topic draws from many international cooperative efforts.

## Author Contributions

This Editorial has been co-authored by the four guest associate editors, TB-R, MM, ÁD-J, and MG. All authors contributed to the article and approved the submitted version.

## Conflict of Interest

The authors declare that the research was conducted in the absence of any commercial or financial relationships that could be construed as a potential conflict of interest.

## Publisher's Note

All claims expressed in this article are solely those of the authors and do not necessarily represent those of their affiliated organizations, or those of the publisher, the editors and the reviewers. Any product that may be evaluated in this article, or claim that may be made by its manufacturer, is not guaranteed or endorsed by the publisher.

